# Reuse of degraded *Pleurotus ostreatus* (Jacq.) P. Kumm. substrate by supplementation with wheat bran. Quantitative parameters

**DOI:** 10.1080/21501203.2016.1168886

**Published:** 2016-04-04

**Authors:** Mª Raquel Picornell Buendía, Arturo Pardo-Giménez, José Arturo de Juan-Valero

**Affiliations:** a Escuela Técnica Superior de Ingenieros Agrónomos de Albacete, Castilla – La Mancha University, Albacete, Spain; b Centro de Investigación, Experimentación y Servicios del Champiñón (CIES), Cuenca, Spain

**Keywords:** Agricultural wastes, edible mushrooms, *Pleurotus ostreatus* (Jacq.) P. Kumm., quantitative parameters, spent mushroom substrate

## Abstract

This research work was conducted in order to investigate the agronomic feasibility of *Pleurotusostreatus* by reusing spent substrates previously in crops of the same mushroom. After the physical and chemical characterization of the substrates, we have evaluated quantitative production parameters in one growing season. As base material, the experiment was arranged in wheat straw (WS) and spent *Pleurotus* substrate (SPS) to generate prepared substrates with the participation of the same, alone, and mixed in different proportions with wheat bran (WB). Unsupplemented SPS, supplemented SPS with 600 g of WB, mixture of WS + unsupplemented SPS, and mixture of WS + supplemented SPS with 600 g of WB, are prepared substrates with biological efficiencies (BE) ranging between 41 and 66% and an excellent unit weight of sporophores harvested. All correlations established among the germination index (GI), earliness (expressed as days to first harvest), yield components and BE were significant and positive correlation coefficients expressed.

## 1. Introduction

The commercial production of mushrooms of the genus *Pleurotus* is along with other species of edible mushroom (*A. bisporus*, and *L. edodes*), a modern and unique economic activity within the field of agronomy, with a remarkable presence both in Spain and around the world. Approximately, 13,500 t of this fungus is produced in Castilla – La Mancha (67 % of the national total) (Pardo et al. 2009). The mushroom growing sector in Spain generates about 5 × 10^5^ t of spent compost, while the EU, as a whole, produces more than 3.5 × 10^6^ t (Pardo et al. 2009; Picornell et al. 2010). This lignocellulosic material called mushroom spent substrate, can be used in various fields of agriculture (animal feed (Zadražil 1980), amendments (Tajbakhsh et al. 2008), substrates of nurseries, nurseries, (Medina et al. 2009)), bioremediation (Faraco et al. 2009), aquaculture, vermiculture, and biofuel (Pathak et al. 2009), but these uses are not enough to take advantage of the high volume generated annually, which accumulates in collection centres located in production areas of Spain. These spent substrates are potential contaminants, not to mention, a waste of energy. Bisaria et al. (1997) emphasize the importance of protein supplementation in substrates with low-nitrogen content, in an organic or mineral way, but in small amounts, being as excess nitrogen can also reduce the degradability of the substrate, adversely interfering with production and biological efficiency. Shin et al. (1997), and Chang and Miles (2004) report that, probably, supplementation with wheat bran (WB) is important to supply the needs of vitamins and other growth factors in the nutrition of different species of fungi Basidiomata.

The aim of this work is the quantitative agronomic evaluation of spent *Pleurotus* substrate (SPS), and its mixture with wheat straw (WS) in different proportions, such as lignocellulosic sources in new growing cycles of *P. ostreatus*, and unsupplemented and supplemented with different doses of WB. The use of the remaining spent mushroom substrate after the cultivation of *P. ostreatus* in new production cycles would be an agronomically viable alternative to using WS partially, which is currently virtually used as a base material exclusively (even more so if you consider the economic problems associated with the use of this cereal farmer’s by-product and the high-market price of WS, especially in drought years). SPS is considered to be environmentally unfriendly, undesirable, and presents a solid waste disposal problem for mushroom growers. If SPS could be easily available at a low cost it could be integrated into new formulations and methodologies, diminish the environmental impact of the waste produced during mushroom cultivation, limit the grower’s dependence on straw, and decrease the environmental impact of its overgrowing accumulation.

## 2. Methods

### 2.1. Analytical methods used for the characterization of materials

The characterization of raw materials and processed substrates was measured according to following parameters: moisture and ash (MAPA 1994), pH and organic matter (Ansorena 1994), total nitrogen (TECATOR 1987; MAPA 1994), C/N ratio, crude fibre (ANKOM 2008), crude fat (ANKOM 2009), nitrogen-free extractives (NFE) (González et al. 1987), cellulose, and neutral detergent‐soluble (NDS) (ANKOM 2005, 2006a, 2006b). Furthermore the exploration of mites (Krantz 1986) and nematodes (Nombel and Bello 1983) was performed.

### 2.2. Growth cycle management

The experimental followed a Balanced Factorial Plan Design 3 × 4, and included 6 replicates for each treatment (randomized block factorial two factors). Source materials used in the preparation of the substrates were SPS remaining after the cultivation of *P. ostreatus*, and the combination of WS with unsupplemented and supplemented SPS with a dose of 600 g, 1.200 g, and 1.800 g of WB. As the control treatment, further two commercial substrates from different locations, we used the WS, unsupplemented and supplemented SPS with the same doses of WB. According to the experimental design, 12 differentiated treatments were generated in the process, moreover of the two corresponding reference commercial substrates. All treatments added CaSO_4_ at 50 g/kg to base material. CaCO_3_ was not added to the four base substrates made up by WS alone, but varying amounts of CaCO_3_ were added to the remaining treatments, depending on the amount of SPS used (20 g/kg of SPS). CaCO_3_ or gypsum was not added to the commercial substrates (Table 1).

**Table 1.** Treatments tested (g/bag) in the experiment.

Treatment	WS	SPS	WB	Gypsum	CaCO_3_
T1	6.000	0	0	300	0
T2	5.400	0	600	300	0
T3	4.800	0	1.200	300	0
T4	4.200	0	1.800	300	0
T5	3.000	3.000	0	300	60
T6	2.700	2.700	600	300	54
T7	2.400	2.400	1.200	300	48
T8	2.100	2.100	1.800	300	42
T9	0	6.000	0	300	120
T10	0	5.400	600	300	108
T11	0	4.800	1.200	300	96
T12	0	4.200	1.800	300	84
T13	Commercially controlled based substrates (A) (6.5 kg/bag)				
T14	Commercially controlled based substrates (B) (6.5 kg/bag)				

WS, wheat straw; SPS, spent *Pleurotus ostreatus* (Jacq,) P. Kumm. substrate; WB, wheat bran; T, treatment; T1, WS 6.000 g; T2, WS 5.400 g + WB 600 g; T3, WS 4.800 g + WB 1.200 g; T4, WS 4.200 g + WB 1.800 g; T5, WS 3.000 g + SPS 3.000 g + CaCO_3_ 60 g; T6, WS 2.700 g + SPS 2.700 g + WB 600 g + CaCO_3_ 54 g; T7, WS 2.400 g + SPS 2.400 g + WB 1.200 g + CaCO_3_ 48 g; T8, WS 2.100 g + SPS 2.100 g + WB 1.800 g + CaCO_3_ 42 g; T9, SPS 6.000 g + CaCO_3_ 120 g; T10, SPS 5.400 g + WB 600 g + CaCO_3_ 108 g; T11, SPS 4.800 g + WB 1.200 g + CaCO_3_ 96 g; T12, SPS 4.200 g + WB 1.800 g + CaCO_3_ 84 g; T13, commercially controlled based substrates (Quintanar del Rey); T14, commercially controlled based substrates (Villamalea).

The first step in the preparation of the tested substrates consisted in chopping and presoaking the WB; later, materials were mixed and the moisture content was adjusted. After these stages in the process, we proceeded to a pasteurizing heat treatment (60–65 °C, 8 h) and progressively decreased to a “seeding” temperature (25 °C) for at least 15 h. Finally, we performed supplementation, “seeding” (dose 30 g/kg mycelium Gurelan H-107) and handmade bagging in Center for Research, Experimentation and Mushroom Services (CIES) pilot plant.

All substrates were packed into transparent polyethylene bags of 29 cm in diameter and a height ranging from 25 to 35 cm, according to the type of substrate, sheltering 6.5 kg approximate of weight. Four holes 2.2 cm in diameter were uniformly drilled over the side surface the bags.

### 2.3. Driving and monitoring of the crop cycle

The total research time was 80 days. The experiment was carried out at the CIES, located in the town of Quintanar del Rey (Cuenca, Spain) in an experimental greenhouse controlled for temperature, substrate temperature, relative humidity, and carbon dioxide concentration and followed the recommended ranges for the variety of selected mycelium and in each stage of development (CIES 2007). Spawned substrates were incubated for 17 days without external ventilation and lighting. During the incubation period, the relative humidity inside the experimental greenhouse ranged between 81 and 96%, while the substrate temperature ranged between 24 and 32 ºC, and the room temperature, between 21 and 28 ºC. After that, we proceeded to allow the development of fruit bodies by ventilation (to keep CO_2_ levels controlled between 0.14 and 0.10%), reduction of the green house temperature (25–16 °C) and the substrate temperature (23–13 ºC) and relative humidity (96–93%) and artificial lighting. These values are close to the microclimatic conditions recommended by other researchers (Pardo et al. 2005b, 2007; García Rollán 2007; Gregori et al. 2008; López-Rodríguez et al. 2008; Gea et al. 2009; Kurt and Buyukalaca 2010).

### 2.4. Evaluation of quantitative parameters

Depending on the level of spawn run time of the substrate by the mycelium and tested contaminations, we established a parameter designated as germination index (GI), on a scale from 0 (no invasion) to 5 (full invasion). Mushrooms were harvested daily at their optimal commercial development. The quantity of “cones” and mushrooms harvested were determined by counting throughout the whole mushroom growth cycle; it was defined as a group of fruit bodies that simultaneously fruited from the same drilled hole in the substrate bag. To evaluate the yield of mushrooms produced daily, each bag was weighed to the nearest gram. The estimated net yield was carried out by weighing the fruit bodies after cutting the unmarketable stipe and calculating the percentage of shrinkage resulting from this operation. Once fruiting occurred, the biological efficiencies (BE) was calculated and expressed as a percentage of the fresh weight of the harvest over the dry weight of the substrate used; the BE was established from the yield provided by each packet, taking into account the charge density of the substrate in the bags and their moisture content. The unit weight of mushrooms (gross and net), expressed in g, was determined from the yields obtained and the quantity of sporophores harvested.

The earliness was established as the time in days since the “seeding” the substrate to the first flush harvested (weighing the daily relative production of the substrate). A flush corresponds to each production cycle that is repeated in rhythm during the harvest. Similarly, we performed a second estimate of earliness considering the total harvest.

Fruiting degree was defined as the ratio between the quantity of cones produced and the quantity of holes made in the bags.

### 2.5. Statistical analysis

To carry out the statistical analysis, we used two software packages: Statgraphics® Plus version 5.1 (Statistical Graphics Corp. 2001), and SPSS® (SPSS 2004). The techniques we employed were descriptive statistics, principal component analysis, variance analysis, and correlation and regression method to evaluate the data.

Differences of *p* < 0.05 were considered significant.

## 3. Results and discussion

### 3.1. Analytical characterization of source materials employees and preparated substrates

The chemical characteristics results of the different source materials, substrates made, and commercially controlled-based substrates are shown in Table 2. According to the results, a higher content of total nitrogen, ash, NFE, and cellulose with a lower crude fibre content and C/N ratio are present in SPS than WS. Meanwhile, WB has a high-total nitrogen content and low-moisture content, ash, crude fibre, and low-C/N ratio. pH values (between 7.11 and 8.36) and moisture content values (between 704 and 743 g/kg) of the substrates tested are within the range normally used in commercial crops.

**Table 2.** Elaborate physicochemical characterization of source materials and substrates used.

		pH	Moisture	Total nitrogen	Protein	Ash	Organic matter	C/N ratio	Crude fibre	Crude fat	NFE	Cellulose	NDS
Base materials	WS	5.85	685	3.5	21.9	76.0	924.0	153.1	391.4	6.9	503.8	407.8	156.8
	SPS	5.50	689	4.8	30.0	89.0	911.0	110.1	190.2	6.6	684.2	453.1	166.0
	WB	6.64	112	23.9	149.4	61.8	938.2	22.8	137.3	30.0	621.5	134.1	353.8
Substrate made	T1	7.70	713	3.6	22.5	199.3	800.8	129.0	355.8	5.8	416.6	324.5	156.3
	T2	8.09	733	7.3	45.3	226.3	773.8	61.9	334.2	10.4	383.8	295.0	165.6
	T3	8.36	743	11.6	72.6	241.1	758.9	37.9	306.6	13.8	365.9	262.8	183.7
	T4	7.11	714	16.4	102.4	218.9	781.1	27.7	261.4	16.5	400.9	225.4	230.4
	T5	7.37	711	4.7	29.6	247.3	752.7	92.2	322.0	5.5	395.6	299.1	186.1
	T6	8.16	725	8.0	49.7	282.5	717.5	52.3	301.4	10.2	356.3	289.4	166.1
	T7	8.28	739	11.2	70.2	230.6	769.4	39.7	287.3	13.6	398.3	270.1	200.1
	T8	8.03	713	13.7	85.7	268.7	731.4	30.9	264.9	16.2	364.6	227.6	210.5
	T9	7.19	704	4.0	25.0	281.6	718.4	104.2	320.0	5.3	368.2	309.0	147.8
	T10	7.81	718	7.8	48.6	234.5	765.6	57.0	322.6	9.9	384.4	317.9	167.9
	T11	8.26	709	10.3	64.6	267.6	732.4	41.1	294.5	13.3	359.9	268.5	179.3
	T12	8.17	715	12.7	79.2	252.1	747.9	34.2	276.7	16.0	376.1	248.8	209.1
	T13	8.08	735	8.2	51.1	70.6	929.4	65.9	448.4	14.1	415.7	389.1	181.3
	T14	7.94	711	8.0	49.9	95.1	904.9	65.7	404.7	12.9	437.4	383.1	169.7
	Average	7.90	720.21	9.1	56.9	222.6	777.4	59.9	321.5	11.7	387.4	293.6	182.4
	CV (%)	5.17	1.73	41.2	41.3	28.7	8.2	50.0	16.3	33.7	6.3	16.9	12.7

WS, wheat straw; SPS, spent *Pleurotus ostreatus* (Jacq.) P. Kumm. substrate; WB, wheat bran; T, treatment; T1, WS 6.000 g; T2, WS 5.400 g + WB 600 g; T3, WS 4.800 g + WB 1.200 g; T4, WS 4.200 g + WB 1.800 g; T5, WS 3.000 g + SPS 3.000 g + CaCO_3_ 60 g; T6, WS 2.700 g + SPS 2.700 g + WB 600 g + CaCO_3_ 54 g; T7, WS 2.400 g + SPS 2.400 g + WB 1.200 g + CaCO_3_ 48 g; T8, WS 2.100 g + SPS 2.100 g + WB 1.800 g + CaCO_3_ 42 g; T9, SPS 6.000 g + CaCO_3_ 120 g; T10, SPS 5.400 g + WB 600 g + CaCO_3_ 108 g; T11, SPS 4.800 g + WB 1.200 g + CaCO_3_ 96 g; T12, SPS 4.200 g + WB 1.800 g + CaCO_3_ 84 g; T13, commercially controlled based substrates (Quintanar del Rey); T14, commercially controlled based substrates (Villamalea). CV, coefficient of variation; NFE, nitrogen free extractives; NDS, neutral detergent-soluble. Results expressed in g/kg dry matter, except pH, moisture (fresh matter) and C/N ratio.

Sánchez (2001) recommended pH values higher than 7 in order to reduce the incidence of *Trichoderma* spp. and other contaminants. This author shows how moisture contents below 500 g/kg and humidity above 800 g/kg will have a negative effect on the growth of *Pleurotus* spp. In this research within the same group of source materials (WS, WS + SPS and SPS), with increasing doses of WB, it increases the total content of nitrogen, protein, and crude fat, while it decreases C/N ratio and cellulose content.

The substrates prepared from WS and WB dose of 1.200 g and 1.800 g (T3 and T4) have higher total nitrogen, protein, and ash content than commercial substrates (T13 and T14). The same situation is shown using as base material WS + SPS and SPS. Ash content was higher than all substrates shown by commercial control, mainly due to the presence of calcium sulphate in the mixtures. The high-nitrogen content in WB (23.9 g/kg) causes the C/N ratio to decrease significantly to increase their share of the substrates, resulting in lower commercial control of all substrates supplemented. Crude fibre and cellulose content of the tested substrates were also inferior in all cases, which showed the commercial control as a consequence of lower organic matter content.

### 3.2. Principal component analysis

This subheading, proceeds to the presentation of the results obtained with the application of multivariate statistical technique of principal component analysis (PCA), according to the physical-chemical characterization of substrates made (Table 3). Total nitrogen content is negatively correlated with C/N ratio, crude fibre, and cellulose content, but it is positively correlated with crude fat content and NDS values. C/N ratio is negatively correlated with crude fat content and total nitrogen, as well as NDS values, but positively to the cellulose content. Crude fat content is significantly negatively correlated with C/N ratio and positively with the total nitrogen content. Additionally, it is also positively correlated to the values of the NDS, with a lower degree of significance. While correlations of cellulose content to total nitrogen, ash content, NDS, and NFE values are negative, correlations of cellulose content with C/N ratio and crude fibre content of substrates are positive.

**Table 3.** Correlation matrix.

	pH									
pH	1.000	Moisture								
Moisture	0.608*	1.000	Nitrogen_T_							
Nitrogen_T_	0.227	0.198	1.000	Ash						
Ash	−0.068	−0.211	0.101	1.000	C/N ratio					
C/N ratio	−0.453	−0.332	−0.910***	−0.149	1.000	Crude fibre				
Crude fibre	0.056	0.151	−0.544*	−0.858***	0.489	1.000	Crude fat			
Crude fat	0.442	0.297	0.925***	−0.179	−0.891***	−0.245	1.000	NFE			
NFE	−0.234	−0.011	−0.206	−0.847***	0.359	0.664*	−0.056	1.000	Cellulose		
Cellulose	−0.005	0.035	−0.662*	−0.764**	0.564*	0.960***	−0.387	0.638*	1.000	NDS
NDS	0.001	0.083	0.885***	0.061	−0.725**	−0.518*	0.770**	−0.023	−0.641*	1.000

NFE, nitrogen free extractives; NDS, neutral detergent-soluble; Nitrogen_T,_ total nitrogen. g/kg dry matter, except pH, moisture (over fresh matter) and C/N ratio. Absolute value of the correlation coefficient between 0.50 and 0.69 (*), from 0.70 to 0.84 (**) or equal to or greater than 0.85 (***).

According to the results, there are three main factors that explain the total variance of the experiment: Factor 1, makes it a 43.03%; Factor 2, does so in a 29.62 %; and Factor 3, 13.44%, assuming between the three, a cumulative variance of 86.09 % (Table 4).

**Table 4.** Total variance explained by each factor.

Factor	Initial eigenvalues		
	Total	% variance	% cumulated variance
1	5.16	43.03	43.03
2	3.55	29.62	72.65
3	1.61	13.44	86.09
4	0.99	8.22	94.31
5	0.34	2.81	97.12
6	0.19	1.61	98.73
7	0.10	0.87	99.60
8	0.04	0.31	99.91
9	0.01	0.06	99.97
10	0.002	0.03	100.00
11	0.00	0.00	100.00
12	0.00	0.00	100.00

In this research Table 5 shows “Rotated Component Matrix” for analytical parameters and experiment factors. In Factor 1, the highest value of the load factor is provided, with a negative sign of the ash content of the substrates tested; this variable for its load factor is followed by: NFE values and crude fibre and cellulose contents. In Factor 2, the total nitrogen content followed by NDS values show higher values of load factor with a positive sign; which are followed by crude fat content (positive sign), C/N ratio (negative sign) and cellulose content (negative sign). Factor 3 is defined by the pH and moisture content of the substrates tested with high-load coefficient values and positive signs.

**Table 5.** Rotated component matrix for analytical parameters and factors.

Analytical parameter	Factor 1	Factor 2	Factor 3
pH	−0.010	0.095	0.919***
Moisture	0.157	0.163	0.754**
Total nitrogen	−0.210	0.946***	0.177
Ash	−0.990***	−0.090	−0.083
C/N ratio	0.276	−0.778**	−0.459
Crude fibre	0.885***	−0.415	0.140
Crude fat	0.057	0.881***	0.386
NFE	0.886***	0.077	−0.316
Cellulose	0.806**	−0.557*	0.078
NDS	−0.150	0.932***	−0.085

NFE, nitrogen free extractives; NDS, neutral detergent-soluble. Extraction method: principal component analysis; rotation method: varimax normalization with Kaiser; the rotation converged in five iterations. Extent of participation, in absolute value, between 0.50 and 0.69 (*), between 0.70 and 0.84 (**), or equal to or greater than 0.85 (***).

### 3.3. Germination index, descriptive statistics and analysis of variance

This study shows in Table 6, the results obtained for the GI of evaluated substrates. Of the 14 different treatments that were generated with various combinations (including commercial substrates), in T8, the mycelium did not develop due to difficulties in germination, with the consequent absence of the production of mushrooms. For the rest of treatments, the greatest inhibition of mycelial growth occurred in the substrates that were supplemented with high doses of WB. The treatments assayed with the highest nitrogen content show a lower GI, including in, treatment T11 (Tables 2 and 6); these substrates have higher a content of protein, crude fat, and NDS, and also the lowest C/N ratio, and crude fibre and cellulose content.

**Table 6.** ANOVA of substrate germination index.

Substrate	Germination index
T1	3.79bc
T2	4.38ab
T3	2.38d
T4	0.63e
T5	4.83a
T6	4.38ab
T7	3.46c
T9	4.88a
T10	4.88a
T11	4.29ab
T12	1.58d
T13	4.54ab
T14	4.54ab
Average	3.73
Fisher F	70.45
Significance level F Fisher	0.00***

WS, wheat straw; SPS, spent *Pleurotus ostreatus* (Jacq.) P. Kumm. substrate; WB, wheat bran; T, treatment; T1, WS 6.000 g; T2, WS 5.400 g + WB 600 g; T3, WS 4.800 g + WB 1.200 g; T4, WS 4.200 g + WB 1.800 g; T5, WS 3.000 g + SPS 3.000 g + CaCO_3_ 60 g; T6, WS 2.700 g + SPS 2.700 g + WB 600 g + CaCO_3_ 54 g; T7, WS 2.400 g + SPS 2.400 g + WB 1.200 g + CaCO_3_ 48 g; T8, WS 2.100 g + SPS 2.100 g + WB 1.800 g + CaCO_3_ 42 g; T9, SPS 6.000 g + CaCO_3_ 120 g; T10, SPS 5.400 g + WB 600 g + CaCO_3_ 108 g; T11, SPS 4.800 g + WB 1.200 g + CaCO_3_ 96 g; T12, SPS 4.200 g + WB 1.800 g + CaCO_3_ 84 g; T13, commercially controlled based substrates (Quintanar del Rey); T14, commercially controlled based substrates (Villamalea). *** *P*-value <0.001. For each column, values followed by different letters are significantly different from each other (*p* = 0.05, Tukey-HSD).

pH of the substrates made is purely basic (pH = 7.11–8.36) (Table 2), which exceeds the desired optimum range for the development of *P. ostreatus* (pH = 5.8 to pH = 6.5) (García Rollán 2007). These values might explain that substrates tested with a higher GI have not had a total colonization.

The application of high doses of WB means an important change in the texture of the substrate, increasing the bulk density and compacting the material, so that it impedes the diffusion of oxygen, leading to the growth and development of the mycelium, which would explain the low-GI values observed.

### 3.4. Quantitative production parameters, descriptive statistics, and analysis of variance

The most noteworthy aspects according to quantitative production parameters are presented in Table 7. The duration of the commercial crop cycle was 80 days, of which 17 days were for the incubation period, except for the substrates made with high doses of WB (T2 to T4, T7, and T12), where this period was shortened. There has been a shortening of the days since the “seeding” until the appearance of the first primordia and until the total induction in treatments that have obtained the lowest GI. This commercial crop cycle stage of edible fungus is highly dependent on environmental conditions and their effects on the temperatures of substrates, of formulations thereof, and their physical and chemical characteristics; the amount of substrate available, the inoculation rate, the distribution of spawn of different species of edible fungi, *Pleurotus*, and finally, types of mycelium or strains (Philippoussis et al. 2001, 2003; Ozcelik and Peksen 2007; Garzón and Cuervo 2008; López-Rodríguez et al. 2008; Hassan et al. 2010), although up to 42 days in *P. ostreatus* (Garzón and Cuervo 2008), 60 days in *P. eryngii,* and 74 days (without induction period) in *V. volvacea* (Philippoussis et al. 2001).

**Table 7.** ANOVA of the quantitative parameters of the experiment.

Substrate	Earliness (days)		Gross yield (g/bag)	Index fructification number cones/hole	Number mushrooms/bag	UW	BE
	1st flush “seeding”	Total “seeding”					
T1	37.0	47.9a	915.8 cd	1.2abc	35.5bc	26.4ab	49.0de
T2	37.3	48.7a	328.7ef	0.7cde	16.3def	16.2b	18.9fg
T3	32.5	32.5b	53.5fg	0.1ef	2.7f	15.7b	3.3gh
T4	14.7	14.7c	11.0 g	0.04f	0.2f	11.0b	0.6 h
T5	34.0	47.0a	1217.5b	1.2abc	32.3bcd	38.9a	65.5c
T6	38.4	53.0a	725.0d	1.2abc	33.3bcd	23.0ab	40.6e
T7	39.8	46.5a	235.5efg	0.4def	11.2ef	25.2ab	13.8fgh
T9	37.0	50.2a	933.7 cd	1.3ab	33.0bcd	28.9ab	48.4de
T10	36.7	48.8a	1155.0bc	1.6a	43.5b	27.5ab	63.1 cd
T11	42.8	52.9a	395.0e	0.8bcd	21.5cde	18.4b	21.4f
T12	10.8	10.8c	4.2 g	0.0f	0.5f	1.4c	0.2 h
T13	32.2	41.6ab	1990.8a	1.6a	84.2a	24.6ab	115.5a
T14	29.1	39.3ab	1778.2a	1.6a	71.2a	25.6ab	94.8b
Average	32.5	41.1	749.5	0.9	29.6	21.7	41.2
Fisher F	1.7	3.3	134.2	26.6	45.1	3.5	124.8
S_L_	0.09^ns^	0.001***	0.00***	0.00***	0.00***	0.001***	0.00***

WS, wheat straw; SPS, spent *Pleurotus ostreatus* (Jacq.) P. Kumm. substrate; WB, wheat bran; T, treatment; T1, WS 6.000 g; T2, WS 5.400 g + WB 600 g; T3, WS 4.800 g + WB 1.200 g; T4, WS 4.200 g + WB 1.800 g; T5, WS 3.000 g + SPS 3.000 g + CaCO_3_ 60 g; T6, WS 2.700 g + SPS 2.700 g + WB 600 g + CaCO_3_ 54 g; T7, WS 2.400 g + SPS 2.400 g + WB 1.200 g + CaCO_3_ 48 g; T8, WS 2.100 g + SPS 2.100 g + WB 1.800 g + CaCO_3_ 42 g; T9, SPS 6.000 g + CaCO_3_ 120 g; T10, SPS 5.400 g + WB 600 g + CaCO_3_ 108 g; T11, SPS 4.800 g + WB 1.200 g + CaCO_3_ 96 g; T12, SPS 4.200 g + WB 1.800 g + CaCO_3_ 84 g; T13, commercially controlled based substrates (Quintanar del Rey); T14, commercially controlled based substrates (Villamalea); UW, unit weight of uncut mushrooms (g); BE, biological efficiency (kg/100 kg of dry substrate); S_L_, F significance level Fisher. ns, no significant difference, *p* > 0.05; *** P-value <0.001. For each column, values followed by different letters are significantly different from each other *(p* = 0.05, Tukey-HSD).

According to the results, as you increase the dose of WB, the gross values or net yield is reduced. Treatments corresponding to the highest doses of bran produced the lowest gross yields, while treatments to commercial substrates produced the highest gross yield. All substrates unsupplemented and supplemented with doses of 600 g of bran, provided higher gross yields. Supplementation did not improve production in any way (except SPS + 600 g bran).

In this study, the best fruiting rates are obtained in unsupplemented treatments with WB and supplemented with 600 g of WB and SPS supplemented with 1.200 g. Supplementation increases with decreasing the rate of fruiting. Gea et al. (2009), worked with *P. ostreatus* grown on specific substrates for this mushroom cultivation, supplemented with commercial protein products. This research offered fruiting index values between 1.21 and 1.57 cones/hole, much higher than values obtained by Pardo et al. (2005b), which ranged from 0.03 cones/hole (grape stalk + “alperujo” 1:1 (v/v)) and 0.75 cones/hole (grape stalk + straw 1:1 (v/v)), including 0.39 cones/hole (pasteurization and thermophilic conditions) and 0.53 cone/hole (benomyl dip and pasteurization), and from 0.43 cones/hole (bag of 5 kg) to 0.50 cones/hole (bag of 15 kg). These same researchers (Pardo et al. 2007), grew *P. ostreatus* on substrates of a varied lignocellulosic nature (cereal straw, kenaf, vine shoot, and “alperujo”) that were subjected to various treatments (pasteurization and thermophilic conditions, moisturizing with benomyl and pasteurization and fermentation semi-anaerobic); they reached values between 0.87 cones/hole, for the combination of straw and “alperujo” 1:1 (v/v) and 1.35 cones/hole for mixing straw and kenaf, and between 0.70 cones/hole, with pasteurization and thermophilic conditions and 1.52 cones/hole in semi-anaerobically fermented substrates. Varnero et al. (2010), with *P. ostreatus* cultivated on WS substrates, eucalyptus chips, slivers of poplar, and mixture of WS and eucalyptus chips; they came to achieve in 1 kg of substrate, a cluster number that ranged from 2.8 (poplar chips) to 5.2 (WS).

The largest quantity of mushrooms was achieved in commercial substrates. SPS achieved better yield compared to WS but not compared to commercial substrates. No supplementation is enhanced by supplementation of 600 g in WS + SPS and SPS. Varnero et al. (2010) in 1 kg of substrate achieved from 3.4 carpophores only (poplar chips) to 18.2 fruiting bodies of the oyster mushroom. Pardo et al. (2005a) show values ranging from 42 mushrooms/package of 15 kg to 80 mushrooms/package of 15 kg, depending on type of mycelium, substrates nature, and treatment of substrates to which they are subjected.

In the respective groups, there were no significant differences in unit weight from a statistical point of view between treatments. Neither supplementation nor the dose was affected; there is a statistically insignificant trend in weight reduction with doses of 1.200 g–1.800 g of WB. Commercial substrates have been overtaken by unsupplemented substrates. Also, there is a certain trade-off between the quantity of mushrooms harvested and the average unit weight. In the study by Pardo et al. (2005a), the average unit weight of fruit bodies was very similar to those obtained in this experiment: between 20.50 and 32.70 g according to nature of substrate, the treatment to which they are subjected to, and the type of mycelium. In a later study, Pardo et al. (2005b) had values between 22 g (grape stalk and cereal straw) and 67 g (grape stalk and “alperujo”); from 29 g (benomyl dip and pasteurization) to 42 g (pasteurization and thermophilic conditions); and between 33 g (bag of 5 kg) and 38 g (15 kg bag). The same researchers (Pardo et al. 2007), in another experiment, depending on substrate tested, reached average unit weight values of the fruit body that ranged from 14.6 g (cereal straw + wine shoot, moisturizing with benomyl and pasteurization) to 25.9 g (cereal straw + “alperujo”, fermentation semi-anaerobic). In another study in Castilla – La Mancha, Gea et al. (2009), they reached figures ranging from 12.41 g (protein supplement) to 14.51 g (unsupplemented control treatment). Varnero et al. (2010) tested eucalyptus and poplar chips as a substrate, and found that they are detrimental to yield components (4.2 and 8.7 g, respectively) compared to WS substrate where the mushrooms come to have an average unit weight of 18.10 g; combining straw and eucalyptus chips remained in intermediate positions, with mushrooms weighing 9.80 g.

Rodríguez Barreal (1987), and Benavides and Herrera (2009) set a standard of BE (50%) below which they don’t recommend growing oyster mushroom commercially; if this value is considered, it could only include: commercial substrates, unsupplemented substrates made of each group of reference, and substrates based on WS + SPS and SPS, both supplemented with 600 g of bran. Except reference commercial substrates, the best results of BE refers to dry substrate in this experiment ranged from 40.6 (T6) to 65.5% (T5); with an increased dose of WB that was accompanied by a decreased BE. Working with *P. ostreatus* and using WS as a substrate, they got BEs similar to this experiment: Upadhyay and Vijay (1991) (65%) and Vogel and Salmones (2000) (64.5 %), when they supplemented with rice bran and flour soy + calcium sulphate, respectively. In research conducted with *P. eryngii* on rice straw, covered by a housing obtained BEs of 47% (Peng 1996). Shan et al. (2004) and Hami (2005), in research based on *P. ostreatus* and using oak sawdust as substrate obtained BE of 64.7 and 69.9 %, respectively. Working with *P. eryngii*, Gaitán-Hernández (2005) using as substrate barley straw and oak wood dust as a supplement, achieved BE of 58%. Tisdale et al. (2006) studied different types of waste for *P. ostreatus* in Hawaii and formulated the substrate as wood chips of different exotic species supplemented with WB. In their study, they obtained BEs between 44.2 and 78.5%. Gregori et al. (2008) achieved BEs of 51% on *P. ostreatus* substrates based on 20% of WB, 10% for waste grain for beer, and 2% of CaCO_3_. These results contradict those reported by Wang et al. (2001) who obtained the highest yields in substrates composed of 45% of WB and 55% of waste grain for beer. With oyster mushrooms, López-Rodríguez et al. (2008) obtained BEs in different agro-industrial residues: calyx of *Physalis* (76.1%), oak sawdust (70%), shell peas (68.6%), and cob cobs (57.8%). Gea et al. (2009) working on specific substrates for growing *P. ostreatus* added supplements prepared from denatured soy flour and other organic protein sources, and reached BEs of 70.6%. With the same species, Naraian et al. (2009) advise to use corncob substrates with chemical supplements (urea and ammonium sulphate) and biological supplements (chickpea flour, soybean meal, groundnut cake, mustard cake, cottonseed cake, and molasses) at low concentrations (0.5 and 2%) to improve BE. Fanadzo et al. (2010) evaluated the BE with various substrates (WS, corn stover and *H. filipendula* and supplements (corn bran and cottonseed) in *P. sajor-caju* and *P. ostreatus*, showing that WS obtained higher BEs (71%) than with corn stover (40%) and *H. filipendula* (35.4%) in the species *P. sajor-caju*. However, corn stover (97%) was most suitable for *P. ostreatus* than WS (45.6%), although the cotton seed supplementation (25%) improved BE in the cultivation of *P. ostreatus* using WS (70.4%). This experiment also showed that supplemented corn bran is not recommended for an increased BE. Also, BEs similar to those in this experiment, but tested on *P. eryngii*, were obtained by Hassan et al. (2010), highlighting the substrate with sawdust (65.2%), while the sugar cane bagasse reached lower BEs (45.7%). These results are consistent with those obtained by Akyuz and Yildiz (2007) (50–73%). Also, Kirbag and Akyuz (2008a) increased their BEs from 48 to 85% when WS was added to millet straw + 10% rice bran, and Kirbag and Akyuz (2008b) from 48.6 to 77.2% when the mixture of WS and cotton was supplemented with 20% of rice bran.

### 3.5. Correlation matrix and “step by step” regression models

According to the results, Table 8 presents the correlation matrix between GI, earliness, and quantitative production parameters, and physicochemical characteristics of the prepared substrates. GI, earliness, yield components, and BE have a significant negative correlation to the total nitrogen content, fat, and NDS values, and a positive correlation to the cellulose contents and crude fibre of the substrates.

**Table 8.** Correlation matrix between germination index, earliness, and production of quantitative parameters, and physicochemical characteristics.

	Germination index	1^st^ flush “seeding”	Total “seeding”	Total quantity of mushrooms	UW	BE
pH	0.010	0.255	0.115	−0.254	−0.352	−0.394
	(0.976)	(0.450)	(0.736)	(0.451)	(0.288)	(0.231)
Nitrogen_T_^1^	−0.838***	−0.617*	−0.737**	−0.827**	−0.749**	−0.836***
	(0.001)	(0.043)	(0.010)	(0.002)	(0.008)	(0.001)
Ash	0.340	0.185	0.245	0.181	0.079	0.131
	(0.307)	(0.587)	(0.468)	(0.594)	(0.817)	(0.701)
C/N ratio	0.560	0.382	0.495	0.686*	0.653*	0.725**
	(0.073)	(0.246)	(0.122)	(0.020)	(0.029)	(0.012)
Crude fiber^1^	0.696*	0.593	0.653*	0.697*	0.584	0.679*
	(0.017)	(0.054)	(0.029)	(0.017)	(0.059)	(0.021)
Crude fat^1^	−0.781**	−0.552	−0.683*	−0.830**	−0.825**	−0.877***
	(0.005)	(0.078)	(0.020)	(0.002)	(0.002)	(0.000)
NFE^1^	−0.188	−0.172	−0.153	0.024	0.214	0.138
	(0.579)	(0.613)	(0.654)	(0.945)	(0.527)	(0.686)
Cellulose^1^	0.852***	0.664*	0.767**	0.898***	0.726**	0.864***
	(0.001)	(0.026)	(0.006)	(0.000)	(0.011)	(0.001)
NDS^1^	−0.826**	−0.729**	−0.794**	−0.771**	−0.536	−0.659*
	(0.002)	(0.011)	(0.004)	(0.005)	(0.089)	(0.027)

UW, unit weight of uncut mushrooms (g); BE, biological efficiency (kg/100 kg of dry substrate); Nitrogen_T,_ total nitrogen; NFE, nitrogen free extractives; NDS, neutral detergent-soluble; ^1^, g/kg dry matter. Results in parentheses indicate statistical significance. No significant (*p* > 0.05) (non *); significant at 95% (0.01 < *p* ≤ 0.05) (*); significant at 99% (0.001 < *p* ≤ 0.01) (**); 99.9% significant (*p* ≤ 0.001) (***).

In the analysis of correlation matrix between GI, earliness, yield components, and BE (Table 9), the existence of significant positively correlated coefficients among them is apparent.

**Table 9.** Correlation matrix between the rate of germination, earliness, yield components, and biological efficiency.

	Germination Index					
Germination index	1.000	1^st^ flush “seeding”				
1^st^ flush “seeding”	0.838***	1.000	Total “seeding”			
	(0.001)					
Total “seeding”	0.929***	0.968***	1.000	Total quantity of mushrooms		
	(0.000)	(0.000)				
Total quantity of mushrooms	0.858***	0.638*	0.779**	1.000	UW	
	(0.001)	(0.035)	(0.005)			
UW	0.773**	0.681*	0.753**	0.782**	1.000	BE
	(0.005)	(0.021)	(0.007)	(0.004)		
BE	0.810**	0.524	0.677*	0.956***	0.863***	1.000
	(0.002)	(0.098)	(0.022)	(0.000)	(0.001)	

UW, unit weight of uncut mushrooms (g); BE, biological efficiency (kg/100 kg of dry substrate). Results in parentheses indicate statistical significance. No significant (*p* > 0.05) (non *); significant at 95% (0.01 < *p* ≤ 0.05) (*); significant at 99% (0.001 < *p* ≤ 0.01) (**); 99.9% significant (*p* ≤ 0.001) (***).

Table 10 shows the “step by step” regression analysis to the physical–chemical properties of substrates, GI, earliness, and quantitative production parameters of the current experiment. Cellulose (positive coefficient) is determined to account for variability in GI and in the quantity of mushrooms. Crude fat content negatively affected the average unit weight and BE. A high-cellulose content of the substrates is necessary for a high GI.

**Table 10.** Models obtained by regressing “step by step”.

Explained variable	Independent variable	Equation	*R*^2^ corrected	SE
GI	PCC	GI = −7.821** + 0.040*** · cellulose	69.60***	0.79801
P2	PCC + GI	P2 = 11.370* + 5.980** · GI	66.90**	5.94030
Nº mushrooms	PCC + QPP (- BE)	Nº mushrooms = −109.230*** + 0.460*** · cellulose Nº mushrooms = −75.870** + 0.803*** · cellulose – 0.423* · CFi	78.50*** 87.10***	7.24998 5.62906
UW	PCC + QPP (- BE)	UW = 43.427*** – 2.037** · CFa	64.60**	6.00518
BE	PCC	BE = 87.472*** – 5.298*** · CFa	74.40***	12.48666

R^2^, determination coefficient (%); SE, standard error of the estimate. Physicochemical characteristics of substrate (PCC): pH (aq. 1:5, w/w), total nitrogen (g/kg, odm), ash (g/kg, odm), C/N ratio, crude fiber (CFi; g/kg, odm), crude fat (CFa; g/kg, odm), nitrogen free extractives (NFE; g/kg, odm), hemicellulose (g/kg, odm), cellulose (g/kg, odm), lignin (g/kg, odm), neutral-detergent soluble (NDS; g/kg, odm), odm, on dry matter. Index germination, earliness and quantitative production parameters (QPP): germination index (GI), days from inoculation to the formation of the first primordia (P2), days from inoculation to the onset of harvest (P4), *n* mushrooms (quantity of mushrooms), average unit weight of uncut mushrooms (UW, g), biological efficiency (BE, kg/100 kg of dry substrate). Significant at 95 % (0.01 < *p* ≤ 0.05) (*); significant at 99% (0.001 < *p* ≤ 0.01) (**); significant at 99.9 % (*p* ≤ 0.001) (***). Regressions include only those whose coefficients accompanying the independent variables are significant, provided that the significance of the model is significant.

Consequently, these formulation based composts degraded by the growth of *P. ostreatus*, could be a low-cost substrate with selective and balanced nutrients for growth and development of oyster mushrooms.

## 4. Conclusions

Unsupplemented SPS, supplemented SPS with 600 g of WB, mixture of WS + unsupplemented SPS, and mixture of WS + supplemented SPS with 600 g of WB, are developed substrates with BE ranging between 41 and 66% with high-average unit weights of carpophores. All correlations established between GI, earliness, yield components, and BE are significant and indicate positive correlation coefficients. Consequently, these gradient based composts for *P. ostreatus* cultivation formulations could be a low-cost substrate with selective and balanced nutrients for the growth and development of oyster mushrooms.
